# Crohn’s Disease Complicated by Retroperitoneal Abscess and Fistula Treated with Single-Incision Laparoscopic Subtotal Colectomy: A Case Report

**DOI:** 10.70352/scrj.cr.25-0081

**Published:** 2025-09-25

**Authors:** Tomohiro Minagawa, Kenji Watanabe, Yusuke Takashima, Toru Watanabe, Takeshi Miwa, Katsuhisa Hirano, Isaya Hashimoto, Kazuto Shibuya, Isaku Yoshioka, Tomoyuki Okumura, Tsutomu Fujii

**Affiliations:** 1 Department of Surgery and Science, Faculty of Medicine, Academic Assembly, University of Toyama, Toyama, Toyama, Japan; 2 Inflammatory Bowel Disease Center, Toyama University Hospital, Toyama, Toyama, Japan; 3 Department of Internal Medicine for Inflammatory Bowel Disease, University of Toyama, Toyama, Toyama, Japan; 4 Third Department of Internal Medicine, University of Toyama, Toyama, Toyama, Japan

**Keywords:** Crohn’s disease, fistula, retroperitoneal abscess, single-incision laparoscopic surgery

## Abstract

**INTRODUCTION:**

Crohn’s disease (CD) can be complicated by complex fistulas and abscesses, and laparoscopic surgery for such cases is difficult. We report a patient with CD who developed a complex fistula and retroperitoneal abscess that were treated using single-incision laparoscopic surgery.

**CASE PRESENTATION:**

A 34-year-old man presented to the hospital after collapsing and losing consciousness while at work. Blood tests showed a white blood cell count of 20000/μL and severe anemia. Abdominal CT showed a right retroperitoneal abscess, which was drained under CT guidance. After gastrointestinal bleeding and an anal fistula were observed, CD was suspected and confirmed by total colonoscopy. The patient was eventually discharged but readmitted 2 months later for abscess recurrence and a colocutaneous fistula. Once the inflammation had improved after 3 weeks of fasting and intravenous antibiotics, the patient underwent single-incision laparoscopic subtotal colectomy, duodenal fistula closure, and seton drainage.

**CONCLUSIONS:**

Single-incision laparoscopic subtotal colectomy can be performed safely to treat CD-related abscesses and complicated fistulas after inflammation has improved.

## Abbreviations

CD
: Crohn’s disease
ECCO
: European Crohn’s and Colitis Organization
IBD
: inflammatory bowel disease
SILS
: single-incision laparoscopic surgery

## INTRODUCTION

Crohn’s disease (CD) is frequently diagnosed at a young age, and between 70% and 80% of patients will require surgery at some point over their lifetime; some even undergo multiple surgeries.^1)^ The reported cumulative rates of redo surgery within 10 years of the initial operation range between 20% and 50%.^2,3)^ Compared with laparotomy, laparoscopic surgery reduces the incidence of adhesion-related readmissions,^4)^ and, therefore, may reduce the incidence of redo surgery in CD patients. In the latest European Crohn’s and Colitis Organization (ECCO) guidelines, laparoscopic surgery is the recommended first-line choice for surgical treatment of CD and as an option for redo surgery.^5)^ Percutaneous drainage is the recommended treatment for CD-related abscess; intestinal resection is usually performed only if needed.^6)^ Any surgery for CD should be performed at a specialized inflammatory bowel disease (IBD) center.^5)^

In Japan, laparoscopic surgery for CD is increasing and being used successfully to treat abscesses and fistulas.^7–9)^ Single-incision laparoscopic surgery (SILS) reportedly provides better cosmetic results than conventional multiport laparoscopic surgery.^10,11)^ Although only a few studies have examined SILS for CD, the results suggest that this surgery is safe and feasible, even in complex cases.^8,9)^ However, most patients in these studies underwent ileocecal or partial ileal resection, so the feasibility of SILS for more difficult procedures is unclear. We present a CD patient with extensive colorectal lesions who developed a retroperitoneal abscess and fistula. After treatment with intravenous antibiotics and percutaneous abscess drainage, we successfully performed single-incision laparoscopic subtotal colectomy, ileosigmoid anastomosis, and seton drainage.

## CASE PRESENTATION

A 34-year-old man presented to the hospital after collapsing and losing consciousness while at work. Blood tests showed a white blood cell count of 20000/μL and severe anemia. His medical and family histories were unremarkable. Abdominal CT revealed a right retroperitoneal abscess, and he was admitted and underwent CT-guided abscess drainage. Gastrointestinal bleeding and a complicated anal fistula were observed during hospitalization. CD was suspected and confirmed after sigmoid colonoscopy revealed mild stenosis, cobblestone appearance, and a rectal ulcer. He was discharged from the hospital 2 months after admission.

**Fig. 1:**
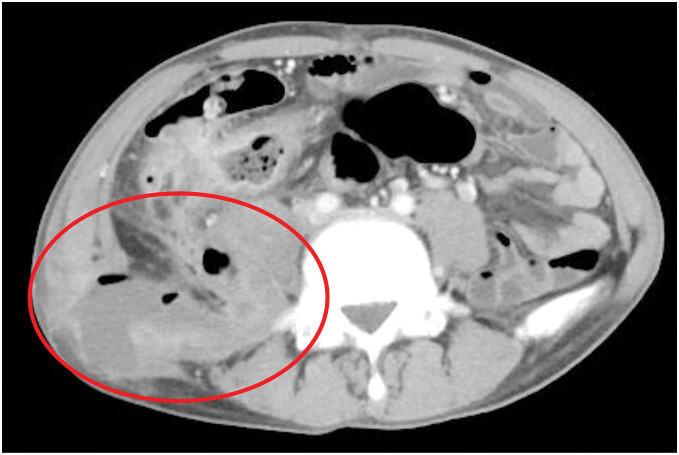
CT findings at admission. The image shows an abscess extending from near the right ilium to the iliopsoas muscle, with recurrence of the previous right retroperitoneal abscess. The circled area indicates the abscess cavity.

During a follow-up outpatient visit, an ascending colon-cutaneous fistula and pus draining from the right side of the abdomen were noted on examination. The patient was then admitted to the hospital, where contrast-enhanced CT showed an abscess around the ilium extending to the right iliopsoas, and recurrence of the retroperitoneal abscess (**Fig. 1**).

**Fig. 2:**
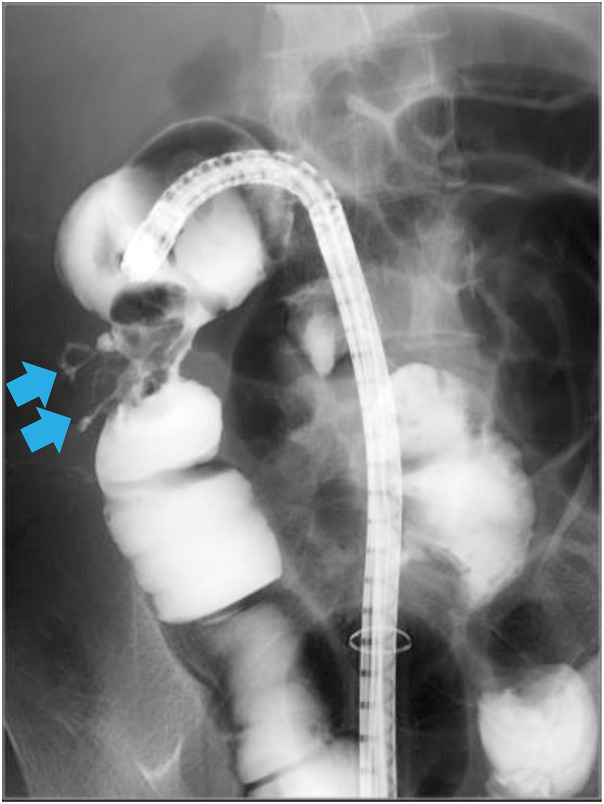
Contrast total colonoscopy examination at admission. The procedure was performed from the ascending colon using a water-soluble contrast medium, which leaked from the ascending colon into the cutaneous fistula. The site of contrast medium leakage into the cutaneous fistula is indicated by the blue arrows.

The patient was then fasted and treated with intravenous cefmetazole 3g daily. After the inflammation had improved, total colonoscopy was performed, which showed a complicated anal fistula, mild rectal stenosis, and severe stenosis in the ascending colon, which was difficult to traverse with the colonoscope. A longitudinal ulcer was observed from the transverse colon to the ascending colon. A selective contrast examination was performed from the ascending colon using a water-soluble contrast medium, which showed contrast leaking from the ascending colon into the cutaneous fistula (**Fig. 2**).

**Fig. 3:**
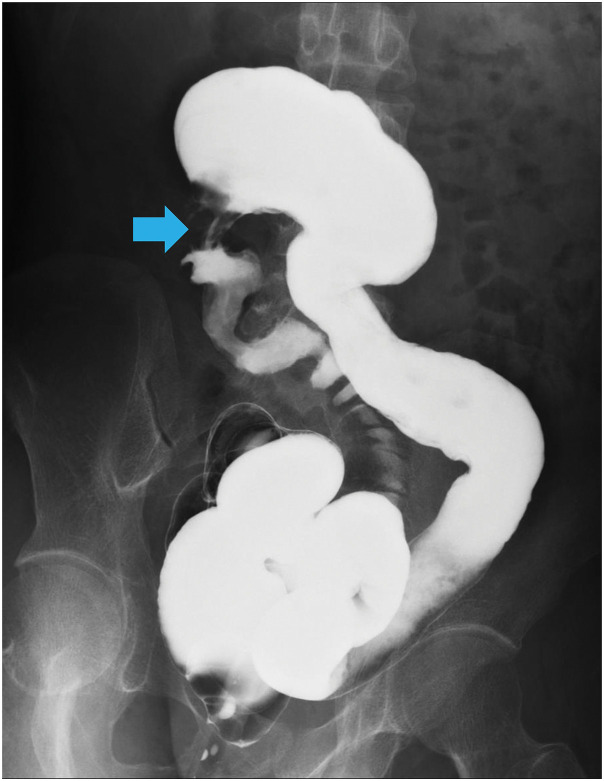
Enema examination just before surgery. There was marked shortening from the cecum to the descending colon, and stenosis was observed in the ileocecal region, with no leakage of contrast medium from the ascending colon into the cutaneous fistula. The blue arrow indicates stenosis in the ileocecal region.

**Fig. 4:**
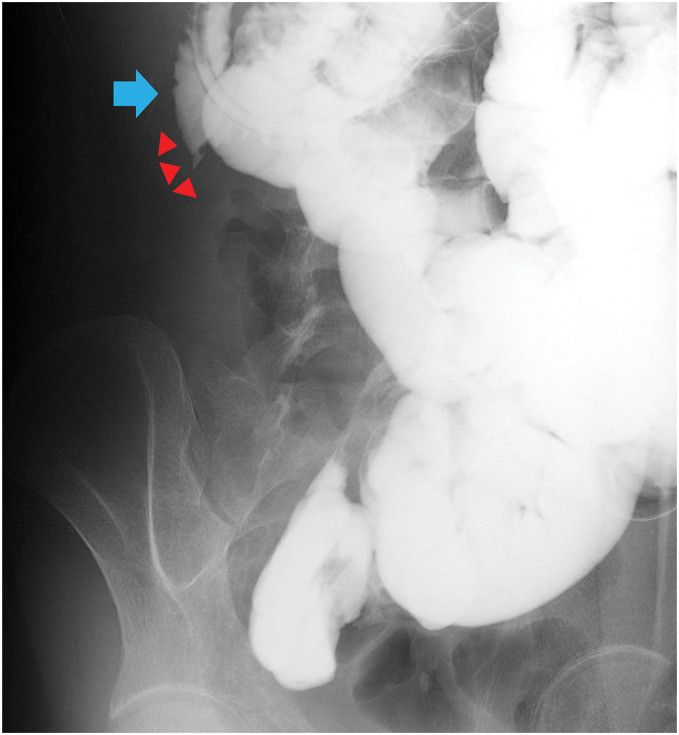
Small intestine contrast examination just before surgery. The image shows a suspected fistula between the duodenum and ascending colon. The blue arrow indicates the duodenum, and the red arrowheads indicate the suspected fistula.

After 3 weeks of fasting and antibiotics, the patient’s abdominal symptoms had improved, and repeat contrast-enhanced CT showed a marked reduction in the size of the abscess cavity. Contrast enema examination showed marked shortening from the cecum to the descending colon as well as stenosis in the ileocecal region; no contrast leaked from the ascending colon into the cutaneous fistula (**Fig. 3**). Contrast examination of the small intestine showed a suspected fistula between the duodenum and ascending colon (**Fig. 4**); no stenosis was seen.

**Fig. 5:**
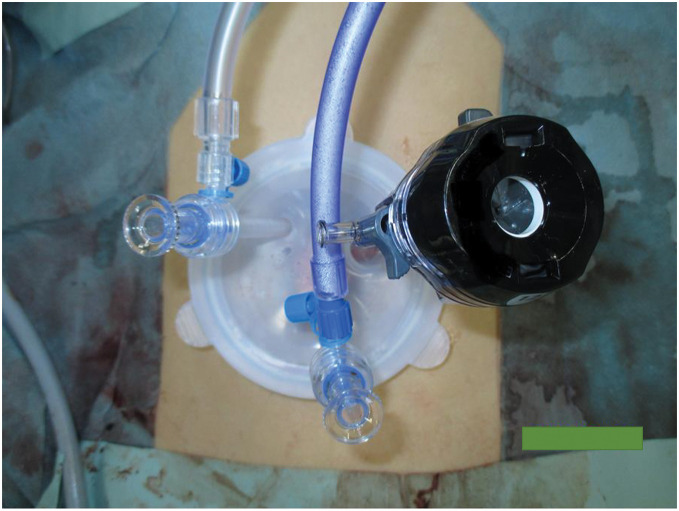
Single-incision laparoscopic surgery. A 4-cm skin incision was made in the umbilical region, and single-incision laparoscopic surgery began with one 12-mm camera port and two 5-mm ports.

**Fig. 6:**
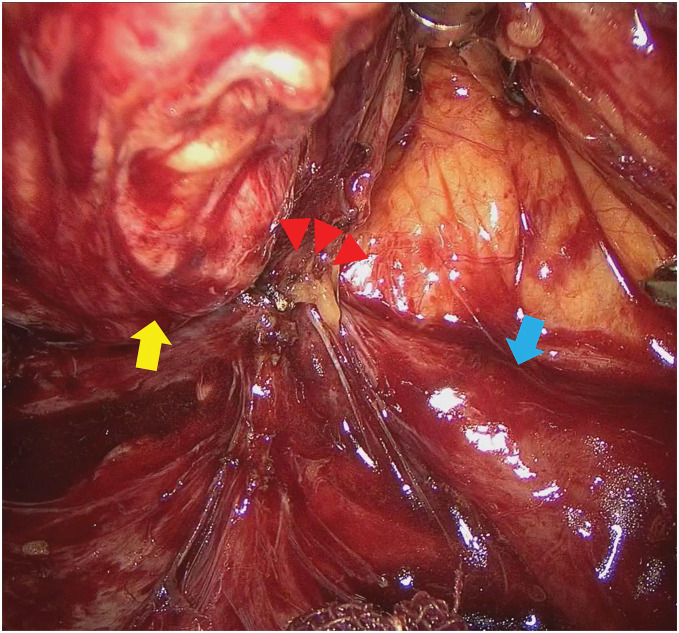
Operative findings showing a fistula between the duodenum and ascending colon. A fistula between the duodenum and ascending colon was identified. The ascending colon is indicated with a yellow arrow, duodenum with a blue arrow, and duodenal fistula with red arrowheads.

**Fig. 7:**
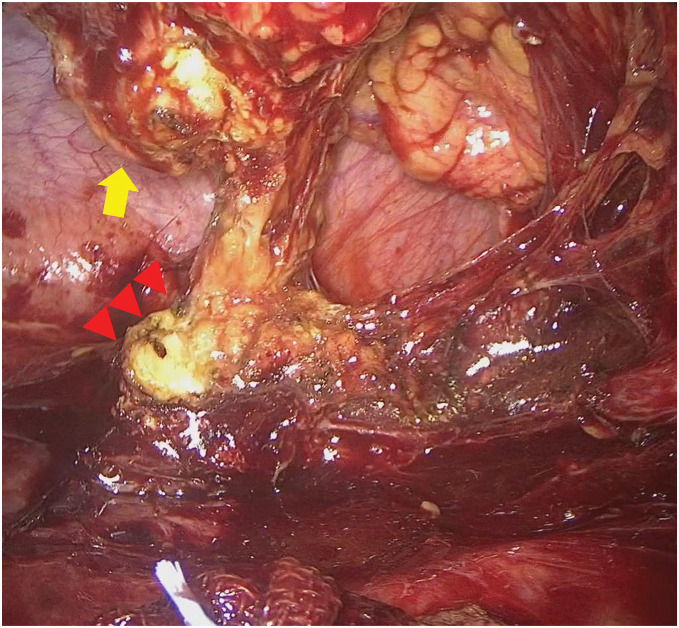
Dissection of the retroperitoneal abscess. Inflammation had improved with treatment, and ileocecal mobilization was relatively easy to perform. The ascending colon is indicated by the yellow arrow, and the abscess cavity is indicated by the red arrowheads.

As surgical treatment, the patient underwent single-incision laparoscopic subtotal colectomy, ileosigmoid anastomosis, and seton drainage. A 4-cm skin incision was made in the umbilical region, a Lap protector and EZ access 100 mm (Hakko, Nagano, Japan) were placed, and one 12-mm camera port and two 5-mm ports were inserted in the same region for surgery (**Fig. 5**). During the operation, mobilization was initiated at the lateral side of the sigmoid colon and proceeded toward the lateral side of the descending colon. The transverse colon, which was markedly shortened, had become incorporated with the greater omentum owing to inflammation, so the hepatic flexure of the transverse colon was first mobilized through the ileocecal region via a retroperitoneal approach. Because of the right retroperitoneal abscess, a fluorescent ureteral stent was inserted into the right ureter to prevent ureteral injury. The fistula between the duodenum and ascending colon was then identified and dissected thoroughly (**Fig. 6**). Ileocecal mobilization was relatively easy to perform and detach (**Fig. 7**). Finally, the greater omentum was incised, the omental bursa was opened on the right side, and the transverse colon was mobilized. There were no obvious lesions in the small intestine.

The mesentery was dissected outside the body cavity. The intestinal tract was resected on the oral side at the end of the ileum and on the anal side at the sigmoid colon in a region without lesions. Then, end-to-side ileosigmoid anastomosis was performed using the Albert–Lembert suture technique. The duodenal fistula was closed simply in 2 layers by trimming the fistula. After the abdominal operation was completed, the anal fistula was treated using 5-site seton drainage. The operation time was 4h and 29 min, and the blood loss was 20 mL. Pathological findings showed marked lymphocytic infiltration in the mucosa from the ileocecal region to the descending colon, with scattered foci extending into the muscularis propria and subserosa. In the subserosa of the ileocecal region, foreign material suspected to be food residue was surrounded by atypical multinucleated giant cells, consistent with peritonitis due to perforation. Mucosal tissue was also found within the muscularis layer of the ascending colon, suggesting diverticula or fistula formation, which may have contributed to the perforation.

On POD 4, a nasogastric tube was placed because of vomiting and paralytic ileus. The tube was removed 3 days later, after the patient’s symptoms had improved and he had defecated. He started to eat on POD 8, and he was discharged on day 16.

## DISCUSSION

CD is commonly diagnosed in teenagers and young adults, many of whom are concerned about the cosmetic aspects of surgical incisions.^12)^ Two randomized controlled trials showed that laparoscopic surgery was associated with significantly shorter incisions and better long-term body image and cosmesis scores.^13,14)^

Previously, laparoscopic surgery was considered a good option to treat simple ileocecal CD cases; more recently, this approach has gained wider use.^7,15–17)^ According to the latest ECCO guidelines, laparoscopic surgery should be the first-line approach to surgical treatment of CD. However, the surgery should be considered carefully in patients with a fragile intestinal tract, severe intra-abdominal adhesions, and complicated intestinal fistulas. Additionally, the laparoscopic approach should be considered carefully in patients who have undergone previous abdominal surgery.^5)^

There are many advantages of laparoscopic surgery: less postoperative pain, improved respiratory function, lower incidence of wound infection, lower risk of incisional hernia, lower risk of postoperative small bowel obstruction, faster recovery of bowel function, shorter hospital stay, and better cosmetic appearance.^13,18–21)^ The lower rates of small bowel obstruction and postoperative adhesions associated with laparoscopic surgery may be advantageous should the patient require redo surgery in the future.^22)^ Most CD patients who undergo redo surgery experience complications, such as abscesses, strictures, and intestinal fistulas.^2)^ These patients also have an increased risk of postoperative complications, such as intra-abdominal sepsis and anastomotic leakage.^23–25)^

The first study of laparoscopic surgery for CD with fistulas and abscesses in Japan reported complication and laparotomy conversion rates of 16%, respectively. The authors concluded that laparoscopic surgery for CD was safe and feasible.^7)^ Another study also reported a high rate of open conversion in patients with severe adhesions or fistulas in the duodenum or vagina; however, patients with small intestine–colon or colon–colon fistulas did not require open conversion.^26)^

The reported median length of the incision necessary for safe extraction from the peritoneal cavity of thickened diseased intestine with CD involvement is 4 cm in SILS.^8)^ This measurement is shorter and more cosmetic than measurements reported in studies of conventional laparoscopic surgery for CD.^8)^ However, SILS has more technical limitations than conventional laparoscopic surgery, such as instrument interference and difficulty achieving access through a small incision.^9)^ Furthermore, Moftah et al. reported a high (15%) open conversion rate in patients with complex CD treated with SILS.^27)^ This is because the anatomical alterations and adhesions caused by fistula formation or previous surgery make laparoscopic surgery difficult and increase postoperative complications.^9)^ However, SILS has been performed safely for CD complicated by fistula or abscess, with no significant difference in the rate of open conversion, incidence of postoperative complications, or blood loss compared with conventional laparoscopic surgery, and with fewer adhesions.^8,9)^ With a 4-cm skin incision, it is possible to place an EZ access 100 mm (Hakko), allowing for a reasonable amount of space to manipulate laparoscopic instruments. Even with the insertion of a 12-mm camera port, operability is not compromised. We routinely use a 10-mm rigid endoscope to minimize interference with the instruments and to ensure better visualization during surgery. By using a 5-mm camera port, there is even less instrument interference. Additionally, the latest 5-mm cameras have been significantly improved, providing high resolution and excellent visualization during surgery. In institutions where such equipment is available, it may be preferable to use the 5-mm camera.

One reason why SILS is possible for complex types of CD is that both physicians and surgeons are involved in the consultation and treatment of patients presenting with abscesses or phlegmon. Therefore, medical treatment or percutaneous drainage of the abscess may be performed preoperatively. Surgery is performed only after inflammation has been controlled and the patient’s nutritional status improves.^9)^ This requires treatment at a specialized IBD center with physicians and surgeons who are well-versed in the treatment of CD. However, the application of SILS still requires careful consideration and extensive experience treating CD patients. Issues that must be considered include the small incision length and the decision regarding whether conversion to multi-port laparoscopic surgery, and if necessary, to open surgery, is required.^8)^ Delayed open conversion has been associated with an increased incidence of postoperative complications^28)^ and the potential risk of CD recurrence.^29)^ SILS for complex CD is safe if performed in a specialized IBD center that can appropriately determine the need for additional ports and open conversion.^8)^ Maeda et al. reported that the indications for SILS are now restricted to simple disease or complex disease not requiring proctectomy or extensive cystectomy.^9)^ Because the surgical procedures required to treat a perforated rectum or urinary bladder are more complicated than those for simple disease, more advanced surgical techniques and improved instruments are needed for such difficult cases.^9)^

Our patient underwent successful SILS to treat a retroperitoneal abscess, ascending colon-cutaneous fistula, ascending colon-duodenal fistula, and marked shortening and inflammation from the cecum to the descending colon. SILS was possible after the abscess was drained and the inflammation improved. Compared with conventional laparoscopic surgery, SILS is associated with better cosmetic outcomes, but surgical safety and long-term prognosis should always take priority. Therefore, SILS should be regarded as one of the treatment options only in carefully selected cases and performed at specialized IBD centers. The ECCO guidelines also recommend percutaneous drainage for intra-abdominal abscesses if possible.^5)^ If drainage is successful, surgery can be avoided, and even if surgery becomes necessary, SILS might be feasible, as in our patient.

While there are no specific reports on the exact number of laparoscopic surgeries or types of procedures required to make SILS feasible for CD, a report on the learning curve for SILS in colorectal cancer showed that for surgeons with sufficient experience in conventional laparoscopic colorectal cancer surgery, the learning curve for right colectomy was between 6 and 15 cases, and for anterior resection was between 13 and 36 cases.^30)^ For SILS in CD, a similar number of cases is likely required. We recommend starting with single-incision laparoscopic right colectomy for simple CD cases, and progressively transitioning to subtotal colectomy or rectal surgery.

With complex CD, SILS can be achieved in selected cases with repeated learning in CD surgeries at specialized high-volume IBD centers, learning characteristic surgical techniques, and performing the procedure under the guidance of an experienced mentor. However, in some complex cases, emergency surgery may be required, and it may be difficult to transfer the patient to a specialized center. In such situations, we believe that conventional multi-port laparoscopic surgery, which is feasible in most facilities, should be the preferred approach. Furthermore, as reported by Simillis et al.^31)^ and He et al.^32)^ stapled side-to-side anastomosis is associated with lower rates of anastomotic leakage and larger anastomotic diameter and is therefore often preferred. According to the ECCO guidelines, stapled side-to-side anastomosis is often preferred from the perspective of reducing postoperative complications, but the guidelines do not specifically recommend one particular technique, instead emphasizing that the choice should depend on the surgeon’s expertise, the location of the CD lesions, and intraoperative findings.^5)^ On the other hand, hand-sewn anastomosis allows for precise control of the suture line and is advantageous for intestines accompanied by inflammation and edema. In CD, where endoscopic examination is important for postoperative follow-up, hand-sewn anastomosis is considered to be closer to a physiological configuration than stapled side-to-side anastomosis and to have good endoscopic passability. In this case, considering the extensibility of the ileal mesentery, the difference in diameter between the sigmoid colon and the ileum, and the residual mild inflammation and edema of the remaining sigmoid colon, we performed a hand-sewn end-to-side anastomosis. There is a report indicating that hand-sewn end-to-end anastomosis outperforms stapled side-to-side anastomosis in terms of long-term function and QOL.^33)^ Therefore, we routinely perform hand-sewn anastomosis, although it remains technically demanding.

Conventional multi-port laparoscopic surgery and hand-assisted laparoscopic surgery are superior to SILS in terms of operability and instrument freedom and are considered particularly useful for complex adhesion dissection and fistula resection.^34)^ On the other hand, the significance of this report lies in demonstrating that SILS can be performed safely and effectively in a patient with complex CD complicated by fistulas and abscesses, with careful case selection and when undertaken by an experienced surgeon. In this case, subtotal colectomy with complete colonic mobilization, closure of a duodenal fistula, drainage of a retroperitoneal abscess, and an end-to-side anastomosis were successfully accomplished through a single incision, followed by 5 seton drainages for perianal fistulas, with a total operative time of 4h and 29 min. This is within the acceptable range compared to a previous report for such cases,^34)^ suggesting that SILS is technically feasible. However, the introduction of SILS requires institutional infrastructure, case selection, and surgeon proficiency, and it is not applicable to all cases. Further accumulation of cases and long-term outcomes is necessary to establish the safety and efficacy of SILS compared with conventional approaches.

## CONCLUSIONS

Single-incision laparoscopic subtotal colectomy can be an option in suitable cases, such as those with CD-related abscesses and complicated fistulas, in which inflammation has been controlled.
